# RapidArc, SmartArc and TomoHD compared with classical step and shoot and sliding window intensity modulated radiotherapy in an oropharyngeal cancer treatment plan comparison

**DOI:** 10.1186/1748-717X-8-37

**Published:** 2013-02-20

**Authors:** Dirk Van Gestel, Corine van Vliet-Vroegindeweij, Frank Van den Heuvel, Wouter Crijns, Ann Coelmont, Bie De Ost, Andrea Holt, Emmy Lamers, Yasmyne Geussens, Sandra Nuyts, Danielle Van den Weyngaert, Tim Van den Wyngaert, Jan B Vermorken, Vincent Gregoire

**Affiliations:** 1 Department of Radiotherapy, University Radiotherapy department Antwerp – UZA / ZNA, Lindendreef 1, 2020, Antwerp, Belgium; 2 Department of Radiotherapy, The Netherlands Cancer Institute – Antoni van Leeuwenhoek Hospital, Amsterdam, The Netherlands; 3 Department of Radiation Oncology, Leuvens Kankerinstituut, Leuven, Belgium; 4 Department of Nuclear Medicine, Antwerp University Hospital, Edegem, Belgium; 5 Department of Medical Oncology, Antwerp University Hospital, Edegem, Belgium; 6 Radiation Oncology Department & Centre for Molecular Imaging and Experimental Radiotherapy, St-Luc University Hospital, Brussels, Belgium

**Keywords:** Head-and-neck cancer, IMRT, Dosimetrical comparison

## Abstract

**Backround:**

Radiotherapy techniques have evolved rapidly over the last decade with the introduction of Intensity Modulated RadioTherapy (IMRT) in different forms. It is not clear which of the IMRT techniques is superior in the treatment of head and neck cancer patients in terms of coverage of the planning target volumes (PTVs), sparing the organs at risk (OARs), dose to the normal tissue, number of monitor units needed and delivery time.

The present paper aims to compare Step and Shoot (SS) IMRT, Sliding Window (SW) IMRT, RapidArc (RA) planned with Eclipse, Elekta VMAT planned with SmartArc (SA) and helical TomoHD^TM^ (HT).

**Methods:**

Target volumes and organs at risk (OARs) of five patients with oropharyngeal cancer were delineated on contrast enhanced CT-scans, then treatment plans were generated on five different IMRT systems. In 32 fractions, 69.12 Gy and 56 Gy were planned to the therapeutic and prophylactic PTVs, respectively. For the PTVs and 26 OARs ICRU 83 reporting guidelines were followed. Differences in the studied parameters between treatment planning systems were analysed using repeated measures ANOVA.

**Results:**

Mean Homogeneity Index of PTV_therapeutic_ is better with HT(.06) followed by SA(.08), RA(.10), SW(.10) and SS(.11). PTV_prophylactic_ is most homogeneous with RA. Parotid glands prescribed mean doses are only obtained by SA and HT, 20.6 Gy and 21.7 Gy for the contralateral and 25.6 Gy and 24.1 Gy for the ipsilateral, against 25.6 Gy and 32.0 Gy for RA, 26.4 Gy and 34.6 Gy for SW, and 28.2 Gy and 34.0 Gy for SS. RA uses the least monitor units, HT the most. Treatment times are 3.05 min for RA, and 5.9 min for SA and HT.

**Conclusions:**

In the treatment of oropharyngeal cancer, we consider rotational IMRT techniques preferable to fixed gantry techniques due to faster fraction delivery and better sparing of OARs without a higher integral dose. TomoHD gives most homogeneous target coverage with more sparing of spinal cord, brainstem, parotids and the lower swallowing apparatus than most of the other systems. Between RA and SA, SA gives a more homogeneous PTV_therapeutic_ while sparing the parotids more, but the delivery of RA is twice as fast with less overdose to the PTV_elective_.

## Introduction

Radiotherapy techniques have evolved rapidly over the last decade with the introduction of Intensity-Modulated Radiotherapy (IMRT) in different forms. The sharp dose fall-off gradient of IMRT permits the administration of a highly conformal and more homogeneous dose to the planning target volume (PTV) compared with conventional and conformal radiotherapy [1]. This allows better sparing of the organs at risk (OARs; e.g. parotid glands, submandibular and minor salivary glands, larynx and swallowing structures such as the constrictor muscles, the base of tongue, the glottic and supraglottic larynx), leading to a decrease in acute and late side effects [2-5].

Despite the widespread use of IMRT in the treatment of head and neck cancer (HNC) there is no level I evidence of superior anti-tumour efficacy and randomised trials are rare. Two phase III studies in stage I/II nasopharyngeal cancer showed a benefit of IMRT in parotid sparing [6,7]. The PARSPORT study of Nutting et al. [8] reported a 50% reduction in late grade 2 or more xerostomia with IMRT compared to conventional radiotherapy in patients with oropharyngeal and hypopharyngeal cancer, of whom 77% of stage III/IV. However, a clear benefit, in terms of local tumour control or survival, of IMRT over the more classical three-dimensional conformal radiation therapy has not been observed so far [9,10]. There are also some concerns about a theoretically higher risk of induction of secondary cancers by IMRT [11,12].

Meanwhile, static beam IMRT has evolved to rotational IMRT based on Brahme’s theory of more degrees of freedom resulting in more conformal dose distributions [13]. These rotational techniques were introduced into the clinic without much evidence of their superiority over classical static beam IMRT [14,15]. There are basically two major ways to perform rotational radiotherapy: cone beam (Volumetric rotational IMRT first introduced as Intensity Modulated Arc therapy (IMAT) by Yu [16], but several alternative approaches have been introduced recently) and fan beam (also referred to as tomotherapy, serial [17] or helical [18]). The treatment of locally advanced HNC patients is technologically challenging and it is not clear which of these IMRT techniques is superior in terms of coverage of the PTV, sparing of OARs, dose to the normal tissue, beam-on time and delivery time. Compared to conventional static beam IMRT, significantly reduced treatment times and total number of monitor units have been reported for RapidArc while maintaining similar dose distributions [19,20]. Bertelsen et al. reported single arc SmartArc in comparison to Step and shoot IMRT, to be at least equivalent in target coverage and conformity while using less monitor units and reducing the beam on time [21]. Van Vulpen et al. reported Helical Tomotherapy to be superior to step and shoot IMRT with a sharper dose gradient and a reduction of the normal tissue complication probability (NTCP) of the parotid glands [22]. Rao et al. found comparable plan quality for Elekta’s VMAT(Volumetric Modulated Arc Therapy) planned with SmartArc, Helical Tomotherapy and fixed field IMRT, with a significant reduction in treatment time with SmartArc at the price of worse sparing of the parotid glands, spinal cord, and brain stem than Helical Tomotherapy [23]. Clemente et al. contested these findings [24]. In complex HNC cases with more than the 4 OARs of Rao contoured, they found VMAT not to provide any distinct advantage compared with Helical Tomotherapy. Oliver’s comparison with RapidArc has shown Helical Tomotherapy to be superior in dose homogeneity at the expense of a longer treatment time and a higher integral dose [25]. Jacob compared treatment plans made in rotational techniques (10 and 25 mm jaw width Helical Tomotherapy and ‘2 full arcs’ RapidArc) with sliding-window (SW) IMRT with leaf widths of 2.5, 5 and 10 mm for nine HNC patients [26]. He concluded that “Helical Tomotherapy plans showed steeper dose volume histograms for the PTV and plans appear to be more conformal and more homogeneous than SW-IMRT plans for all leaf widths and RapidArc plans. The RapidArc technique can reduce beam-on time while maintaining dosimetric quality comparable to that of the SW-IMRT approach”. However, the prescribed dose was only 50 Gy. A recent study by Wiezorek and colleagues compared 7 different ‘machine-treatment planning system’ combinations in a simultaneous integrated boost (SIB) technique to 65.1 Gy or 60.9 Gy [27]. Sliding Window, RapidArc and Helical Tomotherapy showed better target dose homogeneity compared to VMAT and Step and Shoot IMRT. HT Helical Tomotherapy best spared the OARs, results for the other rotational techniques were variable with RapidArc doing worst for the parotids (26.5 Gy mean dose) while being the fastest technique by far.

Most of these studies reported only about a few (the most ‘popular’) OARs and the prescribed doses were not always challenging. Moreover, none of these studies compared with the new TomoHD system, nor has RapidArc been compared with SmartArc in HNC before. The TomoHD™ machine was introduced in the Clinique in 12/2010 by TomoTherapy® to improve the Hi-Art® system. It has a higher maximum gantry speed (12 sec per gantry rotation instead of 15 sec) and also allows for fixed radiation beams (H for helical and D for direct).

In this multi-centric planning study, we will compare the dose distribution in OARs and PTVs obtained with traditional static beam Step and Shoot IMRT of Pinnacle and Sliding Window IMRT of Eclipse with the rotational IMRT techniques of RapidArc planned with Eclipse, Elekta VMAT planned with SmartArc and helical TomoHD using a SIB technique up to 69.12 Gy.

## Material and methods

### Material

For five patients with locally advanced oropharyngeal cancer a contrast-enhanced CT scan with 3 mm slice thickness was acquired in the treatment position with a custom made immobilization mask. Patient characteristics are mentioned in Table 1; examples of individual contours can be found in Additional file 1. Target volumes (primary tumour volume and bilateral elective lymph node regions) and OARs were delineated; all data were sent to the participating centres to be planned on different planning systems.

**Table 1:** Patient characteristics

**Patient**	**Location**	**ICD-O 10**	**Classification TNM/AJCC VI**	**Stage**	**LN + level**	**PTV**_**therapeutic **_**volume (cm**^**3**^**)**	**PTV**_**total **_**volume (cm**^**3**^**)**
1	Base of tongue R	C01	T1N2aM0	IV A	2 & 3 R	104	483
2	Tonsil R	C09	T2N2cM0	IV A	2 bilat & 3 R	233	610
3	Tonsil L	C09	T3N2cM0	IV A	bilat 1,2,3,4	380	955
4	Base of tongue R	C01	T3N2cM0	IV A	1b R & 2 bilat	422	831
5	Tonsil L	C09	T2N1M0	III	2 L	146	563
average						257	688

Bilat = bilateral; LN + = positive lymph node; R = right; L = left; PTV_total_ = PTV_therapeutic_ and PTV_prophylactic_ together; PTV volumes reported in this table are calculated by the Pinnacle treatment planning system.

### Treatment planning

#### Volumes

The gross tumour volume (GTV), the clinical target volume (CTV) and the nearby organs at risk (OARs) were delineated on the Pinnacle 8.0 m planning system. The CTV_69Gy_ (i.e. the CTV_therapeutic_) was defined as the GTV + 1 cm (both for the primary tumour and for lymph node metastases), taking into account that bone, cartilages, ligaments and muscles can prevent tumour spread. The remaining CTV for both the primary tumour (tissue nearby at risk of direct spread) and the bilateral elective lymph node areas (delineated according to Gregoire et al. [28]) were united in the CTV_56Gy_ (i.e. the CTV_prophylactic_). The planning target volumes (PTVs) 69 Gy and 56 Gy were defined as the respective CTVs plus a 3 mm margin with exclusion of the skin. This skin, defined as a 3 mm thick layer under the patient surface, was excluded from the PTV in order to avoid 1) optimization problems with the static beam IMRT systems due to their physical inability to create proper dose in the build-up zone; and 2) overdose to the skin (the so called ‘skin flash’) created by the rotational IMRT systems. The PTV_69Gy_ or ‘PTV_therapeutic’_ was created as a separated volume, i.e. was not included in the PTV_56Gy_ (i.e. the PTV_prophylactic_), to bypass HT’s overlap priority system in which a single voxel can only represent one target volume.

The contoured OARs are listed in Tables 2 and 3. The shoulder was delineated as the humeral head, including the glenohumeral joint, up to the acromioclavicular joint. The top of the lung is defined as the cranial part of the lung above the aortic arc. OARs lying (almost) completely in the PTV were not contoured. A planning risk volume (PRV) of 3 mm was created around the spinal cord and around the brainstem.

**Table 2:** Dose-volume constraints for PTVs and organs at risk

**Target/Organ at risk**	**Median absorbed dose or D50%**	**Mean absorbed dose**	**ALARA**	**Dnear-min or D98%**	**Dnear-max or D2%**
PTV 56	56 Gy		V59.9 Gy	≥ 95% of planned absorbed dose	
PTV 69	69.12 Gy			≥ 95% of planned absorbed dose	≤ 107% of planned absorbed dose
PRV Spinal cord			D2		≤ 50 Gy
PRV Brainstem	≤ 55 Gy		D2		≤ 59 Gy
Parotid gland contralateral		≤ 23 Gy	Mean D, V27		
Parotid gland ipsilateral		≤ 27 Gy	Mean D, V27		
Submandibular gland		≤ 39 Gy	Mean D		
Oral mucosa		≤ 27 Gy	Mean D, V27		
Mandible			V60		
Soft palate		≤ 27 Gy	Mean D, V27		
Constrictor muscles		≤ 55 Gy	Mean D, V20		
Cricopharyngeal muscle		≤ 55 Gy	Mean D, V20		
Base of tongue		≤ 55 Gy	Mean D, V20		
Larynx		≤ 40 Gy	Mean D, V40		
Esophagus superior		≤ 35 Gy	Mean D, V35		
Top of lung			V20		
Inner ear			Mean D, V45		

PTV = planning target volume; PRV = planning risk volume; Vx =% volume receiving x Gy; Dx = dose to x% of the volume; D = dose; ALARA = as low as reasonable achievable.

**Table 3:** Mean volumes of PTVs and OARs calculated by the different planning systems (in cubic centimetre)

		**Pinnacle (SS and SA)**	**Eclipse (SW and RA)**	**Helical tomotherapy (HT)**
PTV 69		257.0	254.2	251.2
PTV 56		431.4	425.3	414.5
* PRV Spinal cord		64.0	63.4	61.8
* PRV Brainstem		53.9	52.8	52.8
* Parotid gland	contralateral	27.6	26.9	26.7
	ipsilateral	26.5	26.0	25.7
* Submandibular	contralateral	7.4	7.1	7.2
* Oral mucosa		51.0	49.1	50.1
* Mandible		67.7	65.7	64.0
* Middle pharyngeal constrictor		1.6	**1.2**	1.5
* Lower pharyngeal constrictor		6.5	**6.1**	6.4
* Cricopharyngeal muscle		2.3	**1.9**	2.3
* Oesophagus (cranial part)		5.3	**4.9**	5.1
* Supraglottic larynx		11.3	**10.3**	11.0
* Glottic larynx		5.1	**4.2**	5.0
* Top of lung	contralateral	438.0	423.5	432.5
	ipsilateral	430.5	415.8	425.1
* Brachial plexus	contralateral	12.7	**11.2**	12.2
	ipsilateral	11.5	**10.3**	11.0
* Inner ear	contralateral	2.5	**2.1**	2.4
	ipsilateral	2.2	**1.8**	2.1
* Brain - PRV brainstem		932.1	914.5	922.2
* Skin near PTV		148.6	145.4	**127.7**
* Eye	contralateral	6.8	6.5	6.6
	ipsilateral	6.5	**6.2**	6.4
* Shoulder	contralateral	161.4	162.0	158.5
	ipsilateral	154.6	156.7	151.8
* Non specified tissue		6473.8	6373.3	6394.0

Bold = more than 5% difference in volume in comparison with Pinnacle; PTV = planning target volume; PRV = planning risk volume.

#### Planning techniques

For each patient a treatment plan was made in the institution where a specific form of IMRT was in use:

1. An inverse step and shoot (SS) IMRT plan was made on a Pinnacle^3^ 8.0 m planning system for an Elekta SL Beam Modulator with 4 mm leave width. Seven equidistant 6 MV beams were used (210°-260°-310°-0°-50°-100°-150°) with a maximum of 80 segments (range 69–79).

2. A sliding window (SW) IMRT plan was made on Eclipse, version 8.6.15, for a Varian CLINAC 2100 C/D linear accelerator with 5 mm leaves in the centre of the field (40 leaves) and 1 cm leaves at the outer 10 cm of the field. IMRT treatment planning was performed using a standard 7 field set-up (220°-300°-340°-20°-60°-140°-180°). The collimators were individually adapted to the PTV and the spinal cord. 6 MV photon beams were used for the anterior fields, while 10 MV photon beams were used for the posterior fields.

3. A RapidArc (RA) treatment was planned for a Varian CLINAC 2100 C/D upgraded with on board imaging (OBI) and RapidArc. The plans were optimized using the Progressive Resolution Optimizer (PRO) 8.6.15 and calculated with Anisotropic Analytical Algorithm (AAA 8.6.15). Each plan consisted of two 6 MV 360° arcs, one clockwise (CW) and one counter clockwise (CCW) of standard 177 control points each. To avoid tongue and groove effects and to improve target coverage and OAR protection, collimator angles were set to 10° (CCW) and 80° (CW*)*[29]*.*

4. An Elekta VMAT treatment was planned using the SmartArc (SA) module in the pre-clinical release v9.0 of Pinnacle^3^ for an Elekta SL20i linear accelerator equipped with a standard MLC with 1 cm leaves not allowing interdigitation. The SA plans were generated using typically a 6 MV dual arc of 356° with advanced leaf travel management and with a final resolution of control points of 4°. The collimator angle was typically set to 20° to avoid tongue-and-groove effects.

5. A Helical Tomotherapy (HT) plan for a TomoHD system was planned on the Tomotherapy planning software version HD1.0 with a maximum of three dose volume histogram control points per volume. A field width of 2.5 cm, a maximum modulation factor of 2.8 and a pitch of 0.287 (to avoid the thread effect [30]) were used. The dose distribution for each beamlet was calculated with a convolution/superposition algorithm. The optimization process used the least mean square optimization method to optimize the objective function.

#### Prescription and constraints

A simultaneous integrated boost technique had to be planned in order to deliver in 32 fractions a dose of 69.12 Gy (2.16 Gy / fraction) to the ‘PTV_69Gy_’ and a dose of 56 Gy (1.75 Gy / fraction) to the ‘PTV_56Gy_’, respecting the prescription guidelines of the International Commission on Radiation Units and Measurements (ICRU) report 83 [31].

The dose to all OARs had to be kept as low as possible respecting the prescription to the PTVs and the constraints for the OARs as mentioned in Table 2. Moreover, the participating centres were encouraged to achieve the lowest dose possible for each OAR, regardless of the prescribed dose (the ‘as low as reasonable achievable’ or ‘ALARA’ principle).

### Data analysis and statistics

#### Reporting

The volume of each structure computed by the different treatment planning systems (TPS) was compared to look for possible bias due to the different volume calculation algorithms of the planning systems.

For the PTVs the homogeneity index (HI, (D2%-D98%)/D50%), the conformity index (CI, V_95%_D_prescribed(body)_/V_95%_D_prescribed(PTV)_), the mean dose, the D_near-min_ (D98%) and the D_near-max_ (D2%) were analysed. The V_59.9Gy_ of the PTV_56Gy_ (= 107% of the prescribed dose) was calculated as a marker for the steepness of the dose gradient towards the PTV_69Gy_. For 30 OARs the mean dose and specific critical doses and volumes were analysed. Organs contoured in less than three patients (due to overlap with the PTV) were excluded from analysis. Finally the beam-on time, treatment time and the number of monitor units were compared.

#### Analysis and statistics

Differences in the studied parameters between treatment planning systems were analysed using the general linear model in the form of a repeated measures analysis of variance (ANOVA). By using a heterogeneous covariance structure in the repeated measures model, we allowed the variance to differ across systems. All included variables were checked for normality. The p-values of group comparisons were adjusted for multiple testing using the false discovery rate (FDR) correction. All hypotheses were tested non-directionally with a p-value of less than 0.05 considered to be significant. All analyses were performed using SAS 9.2 (SAS Institute Inc., Cary, NC).

## Results

### Volumes

Due to differences in the calculation algorithms, the volume of most OARs is smaller when calculated with HT than when calculated with Pinnacle (the median of the mean differences of all OARs was −2.3%, range −1.1% to −14.0%). Compared to Pinnacle, calculated OARs volumes were the smallest with SA’s and RA’s Eclipse (median of mean differences was −4.4%, range 0.9% to −25.3%, with the largest deviations found in the small volumes). The mean volume of the PTV_69Gy_ and the PTV_56Gy_ on Pinnacle was 257 cc (95% CI 83 – 431 cc) and 431 cc (95% CI 330 – 533 cc), respectively. On HT and Eclipse these volumes were 2.3% and 3.9%, and 1.2% and 1.4% smaller, respectively (Table 3).

### PTVs

**Figure 1:**
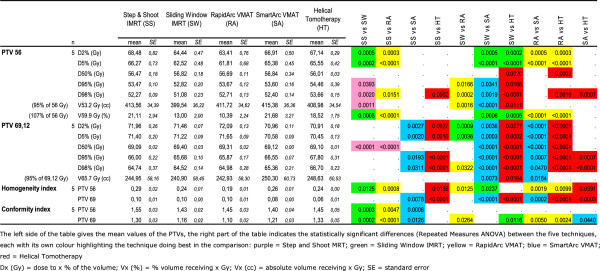
Mean values of PTVs for the different IMRT planning systems.

**Figure 2:**
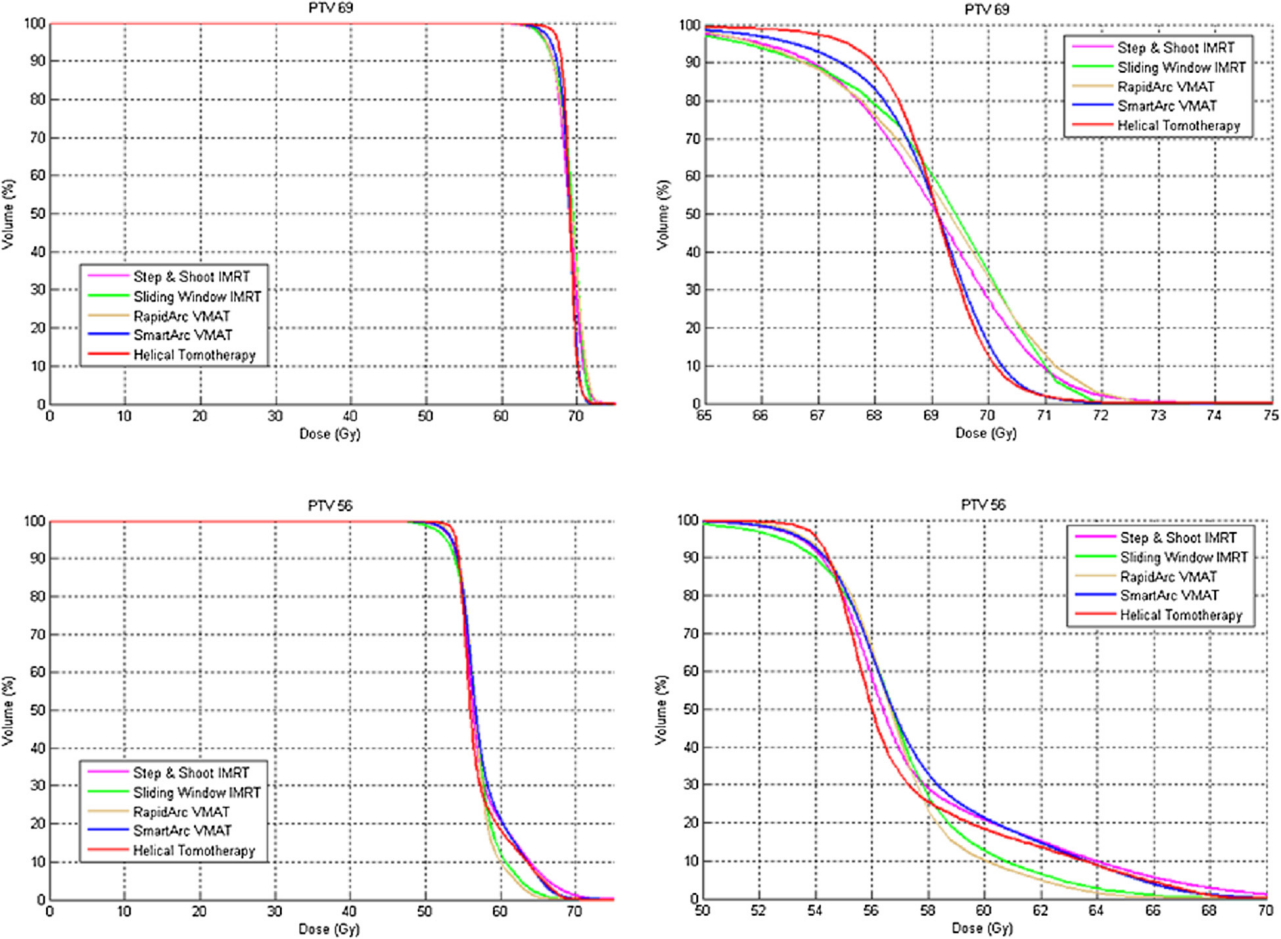
**Dose volume histograms of mean PTV**_**69Gy **_**and mean PTV**_**56Gy**_**.**

An overview of the mean results and corresponding p-values for PTV_69Gy_ and PTV_56Gy_ is given in Figure 1. The corresponding dose volume histograms can be found in Figure 2.

**PTV**_**69Gy**_**:** while the D2% and D50% ICRU 83 guidelines were well respected by all TPS, the D98% guideline was only respected by HT. HT resulted in a statistically significant lower homogeneity index compared to SA, RA, SW and SS. The mean Conformity Index was best for RA and better for SW and SA than for SS and HT.

**PTV**_**56Gy**_**:** compared to the PTV_69Gy_, the D50% guideline was generally less well respected, D98% was only respected by HT. A statistically significant lower homogeneity index was created by RA compared to HT, SW, SA and SS. The Conformity index PTV_56Gy_ was statistically worst for SS.

### OARs

**Figure 3:**
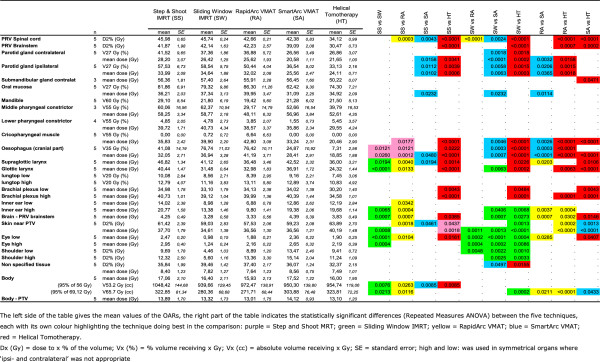
Critical doses and volumes of the different organs at risk for the five planning systems.

**Figure 4:**
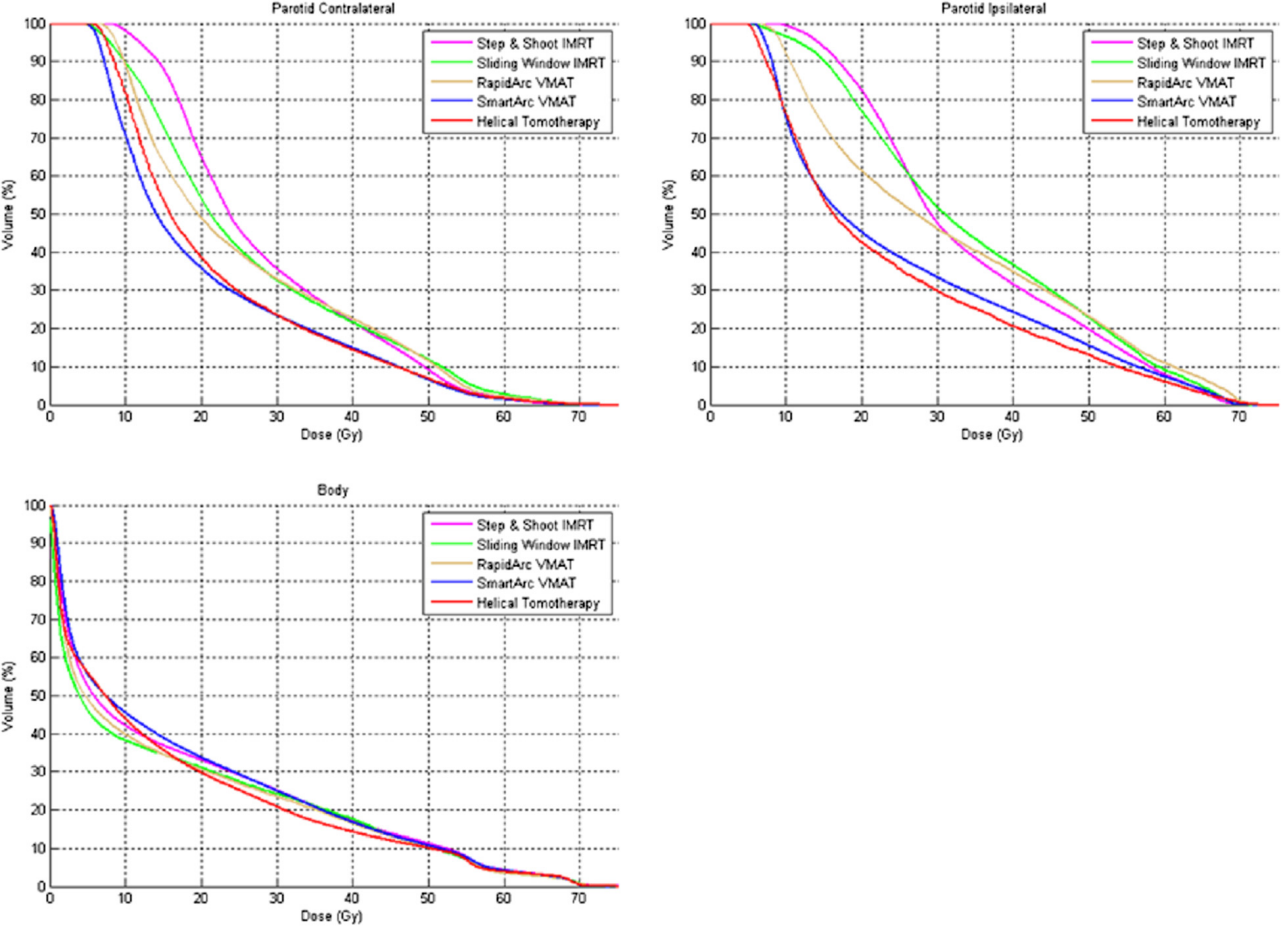
Dose volume histograms of mean values of both parotid glands and total body.

Of the 30 defined OARs four (upper pharyngeal constrictor, base of tongue, soft palate and ipsilateral submandibular gland) were excluded from the analysis because they were contoured in less than three patients due to major overlap with the CTV. For the 26 remaining organs at risk, an extensive list of mean values and of organ specific critical doses and volumes is given in Figure 3. For both parotid glands, for example, only SA and HT fulfilled the prescribed constraints with mean doses of 20.6 Gy and 21.7 Gy for the contralateral gland and 25.6 Gy and 24.1 Gy for the ipsilateral gland, respectively, against RA’s 25.6 Gy and 32.0 Gy; SW’s 26.4 Gy and 34.6 Gy; and SS’s 29.1 Gy and 35.6 Gy (for p-values, see Figure 3). The dose volume histograms of both parotids and the total body can be found in Figure 4.

### Monitor units, beam-on time and treatment time

The results can be found in Table 4.

**Table 4:** Mean beam on time, treatment time and monitor units for the different planning systems

		**Step and shoot IMRT (SS)**		**Sliding window IMRT (SW)**		**RapidArc VMAT (RA)**		**SmartArc VMAT (SA)**		**Helical tomotherapy (HT)**	
	n	mean	*SE*	mean	*SE*	mean	*SE*	mean	*SE*	mean	*SE*
* Beam on time (minutes)	5	1.87	*0.20*	3.74	*0.20*	2.50	*0.00*	4.00	*0.00*	5.94	*0.37*
* Treatment time (minutes)	5	14.01		8.17^*^		3.05		6.00		5.94	
* Monitor Units	5	746	*79*	1125	*59*	415	*18*	608	*24*	5052	*322*

SE = standard error; ^*^ = the irradiation time of one of the sliding window IMRT plans, representative for all sliding window IMRT plans.

## Discussion

Typically, all the IMRT systems are performing quite well with the rotational IMRT techniques not only treating fast but also seeming able to better spare the organs at risk without compromising on the target volume coverage.

However, the results of the small OARs (≤10 cc) should be interpreted carefully as differences in reported volume among TPS may cause some bias. These differences may be due to the calculation algorithm and resolution of the planning systems as reported by Sheng et al. [32]. 

Another concern is the statistical power of a sample size of only 5 patients (each of them judged with the 5 planning systems). The power to detect small differences between systems is low, however, when differences between systems are large and/or the variability within each system is small, one is able to pick up these differences. Also the context of the statistical significances is important. Whenever one system meets a certain constraint where another system fails to reach this constraint, the value of the difference is higher (e.g. parotid glands) than when both systems meet the constraint (e.g. spinal cord, brainstem, constrictor muscles). The value of a difference is low whenever this difference is found for an indices that is not included in the cost function (brachial plexus, eye).

Looking at the PTVs, the D98% ICRU 83 guidelines are only respected by HT, the D2% guideline of the PTV_69Gy_ is respected by all techniques . Based on our results, the coverage of the PTV_69Gy_ is statistically most homogeneous with HT, followed by SA. RA, on the other hand, gives the most homogeneous PTV_56Gy_ and is most conformal for the PTV_69Gy_. The trade-off between the D98% and thus a higher probability of cure on the one hand, and the prescribed mean doses of the OARs nearby resulting in a possible lower local toxicity risk on the other hand, makes this kind of integrated boost planning studies particularly difficult. Moreover, the Conformity Index should be interpreted with caution as a high Conformity Index can not only be explained by ‘uncontrolled’ hotspots outside the PTV but also by a high D98%, pushing a bigger part of the dose gradient outside the PTV.

Although the PTV coverage differences between the plans are often significant, the authors doubt whether these rather small differences will have any clinical impact. The clinical consequences for the OARs, on the other hand, are likely to be more relevant. As the differences in mean doses between the compared systems are quite big for some OARs, the better dose distributions are likely to result into less toxicity. This is the case for both parotids and for the (lower part of the) swallowing apparatus.

Both parotid glands are significantly and probably also relevantly more spared by SA and HT. Numerous authors have pointed out the importance of this parotid sparing in limiting/avoiding late xerostomia as an important factor in the quality of life of the irradiated HNC patient [2-8,33,34].

Another interesting finding is the dose to the swallowing apparatus. Based on our results, the dose to the lower part (cricopharyngeal muscle and cranial part of the oesophagus) of the swallowing apparatus is lowest with HT, SA and SS; while the dose to the glottic larynx is lowest with HT and to the supraglottic larynx highest with SS. These organs have been reported to be important factors in the development of late dysphagia, aspiration and feeding tube dependency [35]. Swallowing dysfunction has been reported to affect the quality of life even more than xerostomia [33]. Moreover, swallowing dysfunction has become a serious threat since the intensification of HNC therapy by the concurrent use of chemotherapy to the radiation or by the use of altered fractionation schedules [34,36].

A lower maximum dose to the spinal cord and the brainstem may be particularly of importance as a substantial number of the patients with locally advanced HNC may develop a loco-regional recurrence within the first two years after primary treatment [37]. Moreover, many HNC patients are predisposed to develop a second primary HNC, from 25% at 5 years to 40% at 10 years [38,39]. These numbers will only increase when patients will live longer as result of a better global HNC management with surgery, IMRT, chemotherapy and biotherapy. For these heavily pre-treated patients high dose IMRT, also in combination with chemotherapy, with or without prior surgery may be the solely remaining curative option [40]. And such high dose IMRT will be less difficult to plan and more efficacious (as higher doses can more safely be administered) when the previous dose to the spinal cord and brainstem was lower, resulting in a higher ‘remaining dose to give’.

Most of the results above are not in agreement with the data of Clemente and Rao as we do find a benefit of HT over SA over SS [23,24].

In contrast to other studies [21,25] and the general opinion, we do not find the rotational techniques to have a higher integral dose as a price for the better sparing of OARs out of the high dose zone. Neither the mean dose to the remaining volume at risk, nor the mean dose to the ‘Body minus PTV’, seems to be higher with the rotational techniques than with the static beam IMRT techniques. In regard to the monitor units (MU), RA uses the least MU and HT by far the most, followed by SW. There has been much debate about a higher number of MUs causing more scatter dose and leaf leakage radiation and thus giving a higher risk of secondary cancers [11,12].

In these technically complicated head and neck patients RA is clearly the fastest IMRT technique, followed by SA and HT HD with comparable treatment times. However, the differences among these rotational techniques seem to be smaller than the differences reported by Wiezorek (i.e. 2 arcs RA vs. 2 arcs VMAT planned with Monaco vs. HT HiArt) [27]. For HT this may be explained by the new HD version used in this study as its maximum gantry speed is faster than the one of the former HiArt system (12 sec / rotation vs 15 sec / rotation). Moreover, all the tested rotational IMRT techniques are found to be faster than the fixed gantry IMRT techniques [19,41]. This may be important, not only for patient comfort but also for tumour control. Of interest in this respect is the observation by Zheng et al. showing that, in a nasopharyngeal carcinoma cell culture experiment, longer fraction delivery times gives less cell kill, probably due to sub-lethal damage repair during the irradiation [42]. However, in HT, every section/slice is treated with high dose rate. It is the sum of all the consecutive sections/slices that takes time. Therefore, Shaikh et al. found the ‘temporal dose delivery pattern’ to be much better for HT than for static beam IMRT [43]. They estimated an increase in the tumor control probability (TCP) of 2-3% with HT compared to the faster 3D conformal RT due to the lower dose rate of the latter. An additional TCP increase of 2-3% due to the shorter total treatment time is suggested for all rotational techniques compared to static beam IMRT. However, only a few studies suggest a benefit this large.

Finally, one has to realise that in the comparison of different IMRT delivery systems and planning systems a lot of variables can influence the results: not only the different calculation algorithms of the different planning systems but also the leaves, the beam angles chosen, the beam energy, the institutional choices made in the trade-off between target volume’s homogeneity, OAR’s sparing and treatment time, the experience of the planner, the time spent on a planning, etc.

## Conclusion

Despite the limitations of a comparison such as used in the present study, we consider rotational IMRT techniques preferable to fixed gantry techniques due to faster fraction delivery times and better sparing of OARs without a higher integral dose. Some of the differences in the OAR doses are quite large so that we do expect a clinical benefit for these rotational techniques.

In this first planning report on TomoHD, the HT system gives the most homogeneous target coverage with more sparing of the spinal cord, brainstem, parotids and the lower part swallowing apparatus than most of the other systems. It also seems to be faster than the former Tomotherapy HiArt version.

In the comparison between RA and SA, SA gives a more homogeneous PTV_therapeutic_ while sparing the parotids more, but RA is twice as fast with less overdose to the PTV_elective_.

## Competing interests

DVG received fees from Accuray/Tomotherapy for occasional lectures for the company. The research of WC is sponsored by Varian. The other authors declare that they have no competing interests.

## Authors’ contributions

DVG participated in the study design and study coordination, prepared patient data sets for treatment planning, performed treatment planning, collected and analyzed data, interpreted data, revised literature and drafted the manuscript. CvV, FVDH, WC, AC, BDO, AH, EL and YG participated in the study design, performed treatment planning, interpreted data and helped draft the manuscript. SN, DVDW and VG participated in the study design, interpreted data and revised the manuscript critically. TVDW interpreted data, helped with the statistical analyses and helped draft the manuscript. JV participated in the study design and study coordination, interpreted data, helped draft the manuscript and revised the manuscript critically. All authors read and approved the final manuscript.

## Supplementary Material

Additional file 1 Images of individual contours of patients. Click here for file
