# Supplementary material for: RapidArc, SmartArc and TomoHD compared with classical step and shoot and sliding window intensity modulated radiotherapy in an oropharyngeal cancer treatment plan comparison
Source: Radiat Oncol. 2013 Feb 20;8:37. doi: 10.1186/1748-717X-8-37 (PMC3599972; doi:10.1186/1748-717X-8-37)
Supplement: Additional file 1 — Images of individual contours of patients. [file 1748-717X-8-37-S1.docx]

Appendix 1: images of individual contours of patients

Patient 1


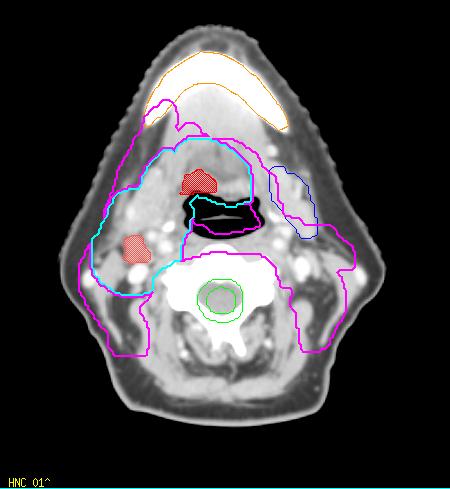


PRV SC

SC

LN+

TU

PTV 69

PTV 56

SMG

Mandible

TU = tumour; LN+ = positive lymph node; PTV 69 = therapeutic planning target volume; PTV 56 = prophylactic planning target volume;

SC = spinal cord; PRV SC = planning risk volume of the spinal cord; SMG = submandibular gland

Patient 2


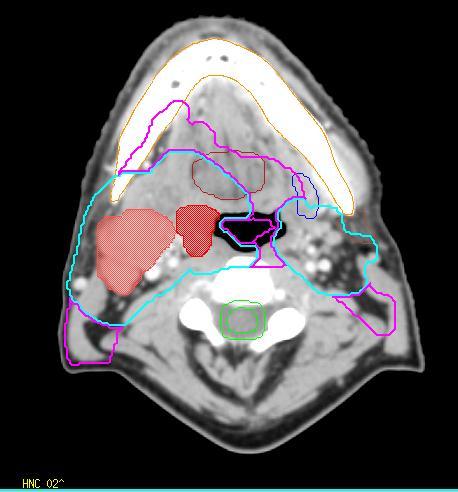


PAR

BOT

SC

PRV SC

PTV 69

LN+

TU

PTV 69

PTV 56

SMG

Mandible

TU = tumour; LN+ = positive lymph node; PTV 69 = therapeutic planning target volume; PTV 56 = prophylactic planning target volume;

SC = spinal cord; PRV SC = planning risk volume of the spinal cord; SMG = submandibular gland; BOT = base of tongue; PAR = parotid gland

Patient 3


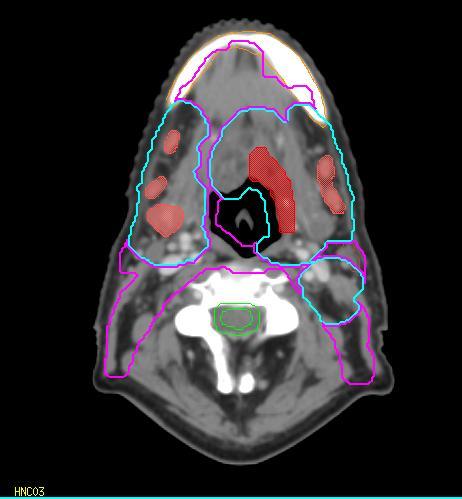


PAR

PRV SC

SC

LN+

LN+

LN+

LN+

TU

PTV 69

PTV 56

Mandible

TU = tumour; LN+ = positive lymph node; PTV 69 = therapeutic planning target volume; PTV 56 = prophylactic planning target volume;

SC = spinal cord; PRV SC = planning risk volume of the spinal cord; PAR = parotid gland

Patient 4


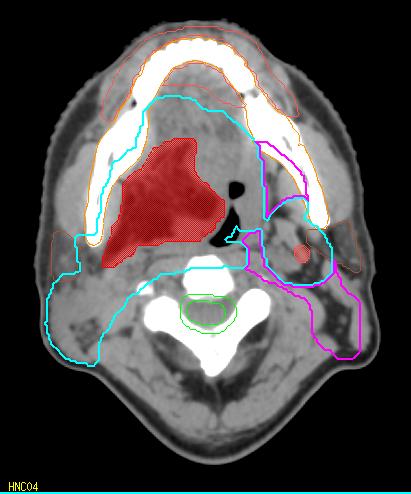


Oral

Mucosae

PAR

PAR

PRV SC

SC

LN+

TU

PTV 69

PTV 56

Mandible

TU = tumour; LN+ = positive lymph node; PTV 69 = therapeutic planning target volume; PTV 56 = prophylactic planning target volume;

SC = spinal cord; PRV SC = planning risk volume of the spinal cord; PAR = parotid gland

Patient 5


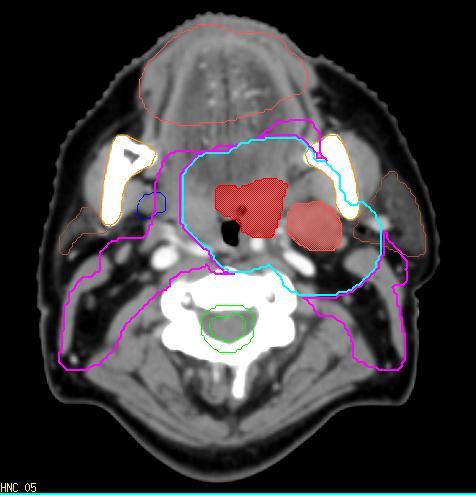


Oral Mucosae

PAR

PAR

SMG

PRV SC

SC

LN+

TU

PTV 69

PTV 56

Mandible

TU = tumour; LN+ = positive lymph node; PTV 69 = therapeutic planning target volume; PTV 56 = prophylactic planning target volume;

SC = spinal cord; PRV SC = planning risk volume of the spinal cord; SMG = submandibular gland; PAR = parotid gland
